# Difference of polymorphism VEGF-gene rs699947 in Indonesian chronic liver disease population

**DOI:** 10.1371/journal.pone.0183503

**Published:** 2017-08-24

**Authors:** Neneng Ratnasari, Siti Nurdjanah, Ahmad Hamim Sadewa, Mohammad Hakimi, Yoshihiko Yano

**Affiliations:** 1 Department of Internal Medicine, Faculty of Medicine Gadjah Mada University/ Dr. Sardjito Hospital, Yogyakarta, Indonesia; 2 Department of Biochemistry, Faculty of Medicine Gadjah Mada University, Yogyakarta, Indonesia; 3 Department of Public Health, Faculty of Medicine Gadjah Mada University, Yogyakarta, Indonesia; 4 Center for Infectious Diseases, Kobe University Graduate School of Medicine, Kobe, Japan; Rosalind Franklin University of Medicine and Science, UNITED STATES

## Abstract

**Background:**

The VEGF gene polymorphism rs699947 related to clinical pathology, mortality, and recurrence of HCC. Few studies mentioned an association between VEGF gene polymorphisms with illness progression in chronic liver disease. We aimed to explore differences of VEGF gene polymorphism rs699947 in chronic hepatitis, liver cirrhosis and hepatocellular carcinoma patients in Indonesian population.

**Methods:**

A cross-sectional study with consecutive sampling and without matching was performed during a 3 years period (2011–2014) at Dr. Sardjito General Hospital Yogyakarta, Indonesia. Blood DNA was sequenced from 123 subjects with chronic liver diseases [39 chronic hepatitis (CH), 39 liver cirrhosis (LC), and 45 hepatocellular carcinoma (HCC)]. 59 healthy subjects also participated. Using isolated VEGF genes for specific primers for rs699947, blood samples were examined by targeting DNA sequences with Applied Bio systems. All data were analyzed using STATA version 11.0 with significance level at *P*<0.05.

**Results:**

The mean of age in HCC and LC subjects were older than in CH and healthy (*P* value <0.05); there were more males in LC, HCC and the healthy groups but not in CH (*P*>0.05). HBV was the dominant etiology in HCC, LC, and CH besides HCV and non HBV-HCV (*P*<0.05). There were significant differences in the SNP -2578 distributions of allele C compared to allele A in all subjects (healthy vs. LC, and HCC; LC vs. CH (*P*<0.05), but no significant difference A>C vs. C>C, and genotypes distribution. Proportion of SNP -2578 A>C vs. C>C CH 1.8:1; HCC 1.4:1; healthy 1.7:1; but its proportion in LC was inversed (1:1.2). Genotype A was low in all subjects (5%-11%). Significant difference of allele distribution was found in healthy vs. LC, and HCC; CH vs. LC. Based on HWE analyses, distribution of allele C was dominant. There were not significant differences in deletion, insertion-deletion at -2547 until -2526, and haplotype (Ht) CCGACCCC (*P*>0.05). The OR analyses of allele and SNP showed that allele A can be a predictor of disease progression in LC to HCC (*OR* 2.26) and healthy to LC (*OR* 1.65); and SNP A>C also can be a predictor in healthy to HCC (*OR* 1.41) and CH (*OR* 1.14).

**Conclusion:**

The occurrence of allele A and SNP A>C VEGF gene (-2578) might predict illness progression from healthy to CH, LC or HCC and LC to HCC.

## Introduction

Angiogenesis and liver vascular damage in chronic liver disease are usually related to progressivity of Liver Cirrhosis (LC) and Hepatocellular Carcinoma (HCC). The occurrence of angiogenesis modulates portal-systemic collateral forming and increases splanchnic vascularization. Stimulation of progressive hypoxia in the hepatocytes can induce the neo-angiogenesis process. Vascular endothelial growth factor (VEGF) is a dominant angiogenesis factor beside other factors such as: cytokine, chemokine, angiopoietin-1, CD40—CD154, and fibroblast growth factor. There are some conditions that may turn normal hepatocytes into malignant cells, such as: cell membrane degradation, hypoxia in center of tumor, mutation of oncogene and tumor suppressor gene. Increasing VEGF in chronic inflammation of hepatocytes can be induced by hypoxia-inducible factors (HIFs), as a consequence of chronic persistent hypoxia [1–5]. Excessive VEGF expression is related to increasing micro vascularization, metastasis and decreasing spontaneous apoptosis. VEGF receptor-2 (VEGFR-2; Flk-1) is a major mediator involved in mitogenic and angiogenic processes during transformation and tumorigenesis [6–8].

The occurrence of VEGF gene polymorphisms can induce either an increase or an inhibition of VEGF secretion, and altered promoter activity. Most researchers agreed that VEGF gene polymorphism is related to clinical pathology, mortality, and recurrence of HCC; even though prevalence of genotype, allele, and site of the polymorphisms are still in doubt. Kong *et al*. (2007) studied a total of 19 allele VEGF gene polymorphisms in HCC, these findings suggest that the -2578 to -1498 region of the VEGF gene showed strong linkage disequilibrium (rs699947; rs1005230; rs12210204; rs833061), and VEGF polymorphisms located at promoter region were associated with HCC progression [9]. Similar study by Wu *et al*. (2009) studied in 7 polymorphisms of VEGF gene of HCC patients (rs699947, rs1570360, rs2010963, rs3024997, rs3025010, rs3025035, rs3025039), the polymorphism rs3025035 located at intron might be a potential genetic marker for HCC recurrence [10]. Wu *et al*.(2013) studied in twelve polymorphisms VEGF gene on HCC and chronic Hepatitis B (CHB) patients, and only six polymorphisms (rs833061, rs1570360, rs2010963, rs25648, rs3025040, and rs10434) were significant different in genotype distribution [11]. The distribution of region identified only two single nucleotide polymorphisms (SNPs) at promoter region (rs833061 and rs1570360) were associated with susceptibility to HCC compared to chronic hepatitis B patients [11]. The occurring of SNP VEGF-634 (rs2010963) G>C located at ‘5UTR in some malignancies but not liver cancer can predict survival and genotype GG acts as a predictor of mortality and recurrence [12]. Our previous study mentioned that genotype GG of the VEGF SNP -634 (rs2010963) was the dominant genotype in severe CLD and HCC compared with healthy [13].

As mentioned to above studies of SNPs VEGF rs69947 located at promoter region, the role of SNP at -2578 A>C, genotype CA, and allele A as predictors of mortality and recurrence in HCC are still debated. The distribution of genotypes based on Kong *et al*. [9] and Wu *et al*. [10] studies revealed that CC genotype (52.9%; 52.2%) is the dominant compare with CA (39.9%; 43.5%) and AA genotypes (7.2%; 4.3%). The result of survival rate analyses in Kong’s study mentioned that genotype AA (-2578) (*HR* 1.69 (1.08–2.63); *P* = 0.020) can be a significant predictor survival compared with genotype CA (*HR* 1.19 (0.93–1.54); *P* = 0.163) [9]. However, the recurrence rate was mentioned in Wu’s study there were no significant different between genotypes [CC (45.8%), CA (50%) and AA (4.2%); *P* = 0.414] and alleles [C (70.8%) and A (29.2%); *P* = 0.401] [10].

According to our knowledge, there were few studies focusing on an association between SNPs VEGF gene with progressivity factors in chronic liver diseases to HCC [11,13]. The aim of this preliminary study is to explore the differences in polymorphism of VEGF-gene rs699947 in chronic hepatitis (CH), liver cirrhosis (LC) and hepatocellular carcinoma (HCC) patients in Indonesian population. The findings of the study can possibly help to predict the occurrence of HCC illness progression from CH and LC.

## Materials and methods

### Patient selection

Inpatients and outpatients with CH, LC and HCC were recruited from Dr. Sardjito Hospital Yogyakarta Indonesia (2011–2014). Healthy subjects were recruited as control subjects. The study was approved by the Medical and Health Research Ethics Committee (MHREC) Faculty of Medicine Gadjah Mada University and the Director of Dr. Sardjito General Hospital. Patients had been screened for eligibility before the study begun. Eligible patients had read the study protocol and signed the informed consent form in order to participate in the study.

The study was reviewed and approved by the Ethics Committee of the Medical and Health Research of the Faculty of Medicine Gadjah Mada University (Reference number: KE/FK/693/EC), based on the ethics principle outlined in the Declaration of Helsinki 2008. A permission letter for the study protocol was submitted by the Director of Dr. Sardjito General Hospital, Yogyakarta, Indonesia.

### Patient eligibility

Patients were considered eligible if they met the following criteria: Adult (>18 years old); Chronic Hepatitis (CH) patients suffering from chronic active disease caused by HBV and/or HCV (based on viral load and liver ultrasound (US)); Liver Cirrhosis (LC) patients diagnosed under clinical criteria, laboratory examinations, and liver-US; HCC patients diagnosed under clinical criteria, laboratory examinations, liver-US or 3 phase MSCT-scan, and fine needle liver biopsy. Patients suffering from upper gastrointestinal bleeding were recruited after the bleeding stopped and were in stable condition. Patients were considered ineligible if they were suffering from at least one of the following conditions: hepatic encephalopathy (grade III or IV); severe sepsis; comorbidity with kidney failure, heart failure, obstructive lung disease, HIV, and/or extra-hepatic solid tumor. Healthy subjects were enrolled in the study with the purpose to determine the sequence of DNA VEGF gene targeted as healthy control specimens. The criteria for healthy control were healthy adult (>18 years old) with normal body mass index, no smoking, and no drinking alcohol; and without any symptoms and signs such as: chronic infectious diseases (HBV, HCV, and HIV), hyperglycemia, and hypertension. All criteria’s were determined by clinical and laboratory examination.

### Study protocol

Patients who met the inclusion and exclusion criteria were recruited after they signed the informed consent form. Baseline data of the patients were recorded and 10 mL blood sample was collected for laboratory examinations [liver function test, thrombosis and hemostasis test using International normalized ratio (INR), blood count, HBsAg, anti HCV, and alpha fetoprotein] during admission at the beginning of the study. One week after the admission, 2 mL blood sample (for DNA isolation) was collected from each patient. DNA isolation from EDTA blood was examined using Wizard^®^ Genomic DNA Purification Kit (Promega^®^, USA; www.promega.com). Polymerase Chain Reaction (PCR) by Platinum^®^ PCR Super-Mix (Invitrogen^®^; Thermo Fisher Scientific Inc.; www.thermofisher.com) was used for DNA amplification. A pair primer was used for seeking targeted sequence of DNA VEGF gene -2578 (rs699947) [14].

Primers of rs699947 (located at promoter, 285 base-pair) are:

(F) 5’-GCCTTAGGACACCATACCGATG-3’;

(R) 5’-GCTGCCCCAGGGAACAAAGTTG-3’

Sequences of targeted DNA VEGF gene were examined using DNA Sanger-Coulson sequencing technique (Applied Bio Systems chromatography^®^; USA; www.lifetechnologies.com).

### Study design

A cross sectional study with consecutive sampling was conducted, without matching of age and gender. Study population was subjects suffering from CH (39 pts.), LC (39 pts.), HCC (45 pts.) and healthy subjects (59 pts.). Independent variables were polymorphisms DNA VEGF gene rs699947 such as: SNP located at -2578, insertion and/or deletion (I/D), and haplotype. Dependent variables were the diseases (CH, LC, HCC, and healthy condition), and confounding variables included age, gender, and etiology of disease. A sample size/power calculation was calculated using formula:

$$N=\frac{1.960^{2}\;\left(PQ\right)}{d^{2}}$$

where a standard normal variate Zα at 5% type 1 error value (two-sided) was 1.960. *P* was an expected proportion in population based on previous study, *Q* was (1 –*P*); and *d* was a precision decided by reseacher, respectively. Based on sample sized equation, the minimal sample sized of HCC was 40.94, and LC or CH was 35.84.

### Statistical analysis

All data were analyzed using STATA software version 11.0, and presented in mean ± standard deviation (SD) if variable distribution was normal or median (minimum; maximum) if the variable distribution was not normal. Bivariate analyses were conducted with *t*-test or the Mann Whitney test for ordinal scales and, chi-square test or the Fisher exact test for nominal scales. Distribution of allele was calculated using Hardy-Weinberg Equilibrium (HWE). The predictive factor was calculated using Odds Ratio (OR). The significance level of variable difference used value *P*<0.05.

## Results

There were 123 subjects matching the study criteria (39 CH, 39 LC and 45 HCC) that also met the inclusion and exclusion criteria, as well as 59 healthy subjects that participated as a control group. Mean of age in HCC (51.53±13.29) and LC (54.72±11.12) subjects were older than in CH (48.26±14.02) and healthy (39.73±13.55) subjects with *P* value <0.05. There were more male subjects than female (109/73; 59.89% vs. 40.11%; *P*<0.05) in HCC 33/12, LC 26/13 and healthy 32/27; but not in CH (18/21). HBV was the dominant etiology in HCC (57.79%), LC (51.28%) and CH (66.67%) besides HCV and non HBV-HCV (*P*<0.05). The history of alcohol drinking was only present in 2 CH subject (5.13%) and 2 LC subject (5.13%). The data of other risk factors such as smoking and family cancer history have not been describe because of diminish information from family and subjects. Distribution of Child Pugh Turcotte (CPT) in LC and HCC was similar (*P*>0.05). Table 1 provides characteristics data concerning study participants.

**Table 1:** Baseline data.

Variables		CH (39)	LC (39)	HCC (45)	Healthy (59)	*P* value
Age, year						0.0001*
	mean ± SD median min; max	48.26±14.02 48 20; 76	54.72±11.12 56 22; 72	51.53±13.29 52 28; 80	39.73±13.55 40 21; 69	<0.05^+^ >0.05^$^
Gender, n(%)						0.046^#^
	male female	18 (46.15) 21 (53.85)	27 (69.23) 12 (30.77)	33 (73.33) 12 (26.67)	32 (54.24) 27 (45.76)	
Etiology,n(%)						
	hepatitis B hepatitis C hepatitis B+C non-hep B+C	26 (66.67) 13 (33.33) 0 0	20 (51.28) 8 (20.51) 1 (2.56) 10 (25.64)	26 (57.78) 5 (11.11) 0 14 (31.11)	-	<0.001^†^
	Alcohol	2 (5.13)	2 (5.13)	0	-	<0.05^#^
CPT, n(%)						
	A B C	-	10 (25.4) 22 (56.41) 7 (17.95)	17 (37.78) 21 (46.67) 7 (15.56)	-	0.536^#^

CH, chronic hepatitis; LC, liver cirrhosis; HCC, Hepatocellular carcinoma; CPT. Child Pugh Turcotte; SD, standard deviation; min, minimum; max, maximum; non-hep B+C, non hepatitis B and C.

* Kruskal-Wallis test.

^#^ Chi^2^ test.

^†^ fisher’s exact test; significant value of *P*<0.05.

^+^ different between healthy vs. LC-HCC-CH; and CH vs.LC.

^$^ different between LC vs. HCC and CH vs. HCC.

**Fig 1:**
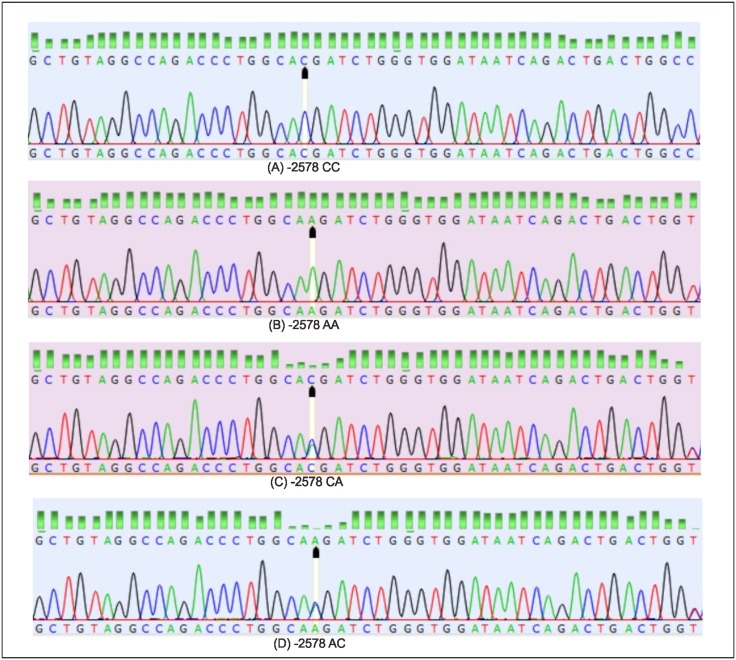
The single nucleotide polymorphism (SNP) VEGF gene located at -2578 (promoter (black arrow). (A) Genotype CC; (B) Genotype AA; (C) Genotype CA, and (D) Genotype AC.

**Fig 2:**
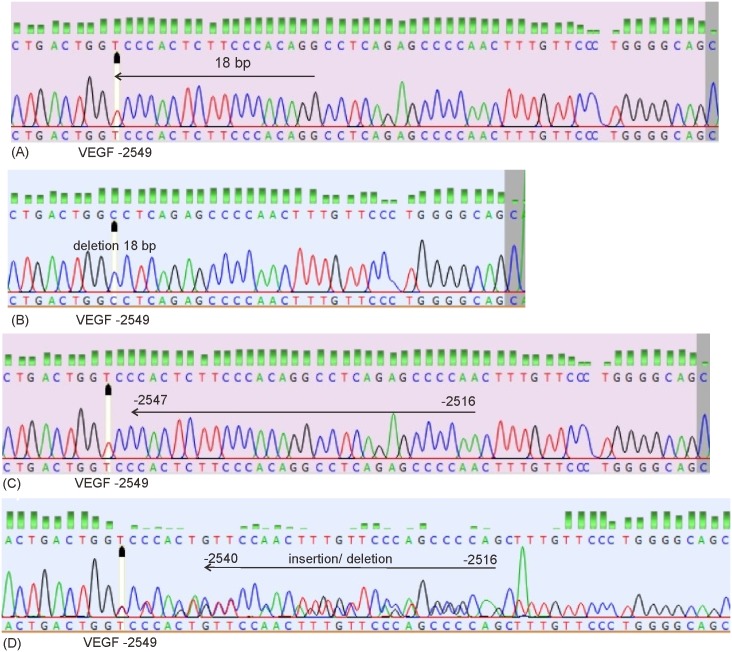
The 18 base-pair deletion VEGF -2549 and insertion/deletion VEGF -2547 until -2516 located at promoter. (A) A normal sequence with location of 18 base-pair deletion at -2549 (black head arrow); (B) A sequence of 18 base-pair deletion -2549; (C) A normal sequence with location of insertion/deletion -2547 until -2516; (D) A sequence of insertion/deletion -2540 until -2516.

Using a pair primer of rs699947 [F: 5’-GCCTTAGGACACCATACCGATG-3’; R: 5’-GCTGCCCCAGGGAACAAAGTTG-3’], the median (minimum; maximum) length of sequence is 251 base-pair (217 bp; 264 bp), GC content 54.95% (53.27%; 56.55%), AT content 45.04% (43.44%; 46.72%), and 97.24% in very good trimming. Based on sequence results, there were single nucleotide polymorphisms (SNP) A>C at -2578, deletion 18 bp located at -2549, variation of insertion-deletion (5 bp– 23 bp) located at -2547 until -2526, and haplotype CCGACCCC. Figs 1 and 2 show the features of the VEGF-gene polymorphisms in rs699947.

There were significant differences in the distributions of allele C compared to A in all subjects (*P*<0.05), but no significant difference in SNP A>C vs. C>C (*P*>0.05) and genotypes distribution. Proportion of SNP -2578 A>C in CH-HCC-healthy subjects was more frequent than C>C (CH 1.8:1; HCC 1.4:1; healthy 1.7:1) but its proportion in LC was inversed (1:1.2). Genotype A was lowest in all subjects (5%-11%) and genotype CA (40%-60%) and CC (30–59%) were similar. Significant difference of allele distribution was found in healthy vs. LC, and HCC; LC vs. CH (*P*<0.05). Distribution of alleles based on HWE showed that allele C (65%-77%) was the most dominant followed by 26%-36% allele CA and 18%-27% allele C, but no significant difference was found between groups. Approximately 90% sequence showed deletion [18 base-pair (bp)] located at -2549, insertion deletion at -2547 until -2516) and 80% - 90% without haplotype (Ht) CCGACCCC. However, there were not significant differences in deletion distribution in rs699947 sequence, insertion-deletion at- 2547until -2526, and Ht CCGACCCC (*P*>0.05). Table 2 displays data concerning polymorphism of VEGF-gene rs699947.

**Table 2:** Polymorphisms of VEGF-gene rs699947.

		CH (39)	LC (39)	HCC (45)	Healthy (59)	*P*
SNP-2578, n(%)						0.384^@^
	C>C A>C	14 (34.90) 25 (65.10)	21 (56.41) 18 (53.59)	19 (42.2) 26 (57.8)	23 (38.98) 36 (61.02)	
Genotype, n(%)						0.848^$^
	CC CA/AC AA	14 (35.14) 23 (59.46) 2 (5.41)	21 (53.85) 16 (41.03) 2 (5.13)	18 (40.91) 21 (47.73) 5 (11.36)	23 (38.98) 30 (50.85) 6 (10.17)	
Allele, n(%)						0.010^#^
	Allele C Allele A	24 (61.54) 15 (38.46)	32 (82.05) 7 (17.95)	30 (66.67) 15 (33.33)	29 (49.15) 30 (50.85)	
HWE (%)						0.665*
	Allele C Allele A Allele CA	65.34 27.43 34.87	76.92 17.89 25.64	64.44 22.92 35.55	64.41 22.92 35.59	
Insertion-deletion, n(%)						0.857^$^
	Del 18bp (-2549) ID(-2547;-2516) Normal	17 (43.59) 18 (46.15) 4 (10.26)	18 (46.15) 18 (46.15) 3 (7.70)	19 (42.22) 22 (48.89) 4 (8.89)	32 (55.17) 21 (36.21) 5 (8.62)	
Ht CCGACCCC n(%)						0.465^$^
	Yes No	5 (12.82) 34 (87.18)	7 (17.95) 32 (82.05)	3 (6.67) 42 (93.33)	8 (13.56) 51 (86.44)	

CH, chronic hepatitis; LC, liver cirrhotic; HCC, hepatocellular carcinoma; SNP, single nucleotide polymorphism; HWE, Hardy-Weinberg Equilibrium; Del, deletion; ID, insertion + deletion; Ht, haplotype.

^@^ Chi^2^ test, no significant different in all subjects A>C vs. C>C (*P*>0.05).

^#^ Chi^2^ test, significant different in: healthy vs. LC, and HCC; CH vs. LC (*P*<0.05); but not significant in HCC vs. LC, and CH; healthy vs. CH (*P*>0.05).

* Chi^2^ test.

^$^ Fisher’s exact test.

The results of the Odd ratio (OR) analyses for alleles and SNP showed that allele A can predict the disease progression in LC to HCC (*OR* 2.26) and healthy to LC (*OR* 1.65). SNP A>C also can predict the disease progression in healthy to HCC (*OR* 1.41) and CH (*OR* 1.14). Table 3 displays the OR analyses for alleles and SNP.

**Table 3:** The Odds Ratio analyses of SNP -2578 in all subjects.

	**HCC–healthy**		**HCC–LC**		**HCC—CH**	
	***OR***	**95%CI**	***OR***	**95% CI**	***OR***	**95% CI**
Allele A	0.49	0.214–1.085	2.26	0.817–6.693	0.80	0.323–1.986
Allele C	ref		ref		ref	
A>C	1.41	0.643–3.121	1.59	0.666–3.828	0.77	0.312–1.87
C>C	ref		ref		ref	
	**LC- healthy**		**LC–CH**		**CH–healthy**	
	***OR***	**95% CI**	***OR***	**95% CI**	***OR***	**95% CI**
Allele A	1.65	0.722–3.818	0.35	0.118–0.998	0.61	0.262–1.385
Allele C	ref		ref		ref	
A>C	0.55	0.239–1.109	0.48	0.191–1.204	1.14	0.491–2.682
C>C	ref		ref		ref	

OR, odd ratio; HCC, hepatocellular carcinoma; LC, liver cirrhosis; CH, chronic hepatitis; and CI, confidence interval.

95% confidence limits testing exclusion of 0 or 1, as indicated *P*-values < 0.05.

## Discussion

This study recruited HBV and HCV-infected subjects who were at risk of advanced liver disease (LC and HCC). These hepatitis B and C patients underwent or planned for anti-viral therapy. Overall, HBV is the most frequent etiology (58.54%), followed by HCV (21.14%) and non-HCV/HBV (28.57%) in LC-HCC. HBV potentially induces HCC directly via HBx protein that has a potential exon of retinoic acid receptor B and cyclin-A2 gene integrated to host DNA (usually regulator proto-oncogene and tumor suppressor genes). Production of surface protein (*S* and pre-S genes) in endoplasmic reticulum can indirectly induce chronic inflammation, regenerative hyperplasia, deregulation of transcription, and hepatocyte progression to carcinoma. Spontaneous mutation and exogenous damage of hepatocyte DNA may occur as the consequences of the integration of mutated DNA virus to host DNA and this condition can also occur in other hepatocytes (‘daughter cells’) [14–17]. Similar with HBV, HCV can induce carcinogenesis via HCV-core protein. The mechanism of carcinogenic induction is related to the replication of positive polarity of single-stranded RNA. RNA virus cannot integrate in the chromosomal DNA host in the hepatocyte, but directly HCV has carcinogenic properties even if the hepatocytes have not seen chronic inflammation and/or cirrhosis. Another condition occurs where activity of hepatocytes in chronic inflammation are related with increasing host DNA proliferation of specific hepatocytes (oncogene and growth regulator genes) [14–15, 18].

Most SNP gene VEGF affect the VEGF excretion and secretion, which is located at promoter, 5‘UTR, intron, and 3’UTR, consequentially influencing the activity of RNA transcription (initiation, elongation, enhancer and termination) and RNA translation. Integration of HBV and HCV in human occurred in the promoter gene. However, the virulence of HBV and HCV also play an important role in increasing activity of hepatocytes necro-inflammation. Previous studies focusing on mutation HBV (dominant sub-genotype B3) in Indonesian populations revealed that there are mutations at Enhancer II and basal core promoter (BCP; C1638T, C1753V, T1762, A1764) and precore (PC; 1896). These mutations may result in increased viral load [19–20].

SNP gene VEGF rs699947 in promoter is at -2578. Polymorphisms in the VEGF promoter are associated with susceptibility to hepatocellular carcinoma by altering promoter activity [11]. In this study, the frequency of SNP-2578 A>C among healthy, HCC and CH were lower than C>C and those among LC subjects A>C higher than C>C (*P*>0.05), but allele A distributions were significantly different with allele C (*P*<0.05) in the healthy group compared with HCC, LC, and CH subjects; and CH compared with LC subjects. According to our knowledge, there are two studies mentioning the location of SNPs VEGF at -2578. However, these studies were performed in HCC subjects only [8–9]. These studies found that the frequencies of genotype and allele of rs699947 (-2578) and were similar to the findings in this study. Polymorphism rs699947 (-2578) has some correlation with mortality risk in Kong’s study, those genotype CA and AA in SNP VEGF -2578 may increase mortality risk in HCC subject (*HR* 1.19; *HR* 1.69) [9].

All subjects were dominated by allele C, and its frequency was higher among LC, HCC and CH subjects than healthy subjects. Two previous in vitro study were reported that -2578 CC genotype and -2578 C allele correlated with a higher VEGF production than the A allele and non CC genotype [21–22]. The frequency of allele A among LC was lower than CH and HCC. This finding means that increases of allele A may cause an increase in VEGF activity due to induction by excessive hypoxia of hepatocytes such as in necro inflammation (chronic active hepatitis) and carcinogenesis (HCC). The processes of hypoxia and angiogenesis can advance together with fibrogenesis immediately after hepatocyte injury, progressively [23–24]. Allele A in LC subjects was lowest (17.89%), meaning that neo-angiogenesis activity progresses more slowly than in HCC and chronic hepatitis subjects. This finding may be due to the fact that the number of hepatocytes in LC is relatively small and will be replaced by fibrotic cells. Activity of proangiogenesis mediators (VEGF, PDGF and PlGF) is used in LC to maintain hyperdynamic splanchnic circulation in portal hypertension [3].

The deletion of -2549 (18 bp) and insertion-deletion of -2547 to -2516 in rs699947 sequencing in this study occurred in some subjects; however, no significant difference was found among subjects. Frequencies of the deletion of -2549 and insertion-deletion in HCC were similar to Kong’s study (deletion: 42.22% vs. 52.9%; insertion/deletion: 48.89% vs. 39.9%) [9]. VEGF is a highly polymorphic gene with potential functional SNPs in cancer risk. Some widely studied SNPs, i.e., -2578 A>C (rs699947), -2549 insertion/deletion polymorphism, -460 T>T (rs833061), -1154 A>G (rs1570360), -634 G>C (rs2010963), +936 T>C (rs3025039), +1612 G>A (rs10434), are reported to be associated with multiple cancers but with controversial results. The most of SNPs VEGF gene were in promoter, linkage disequilibrium with each other, combined effects of these SNPs may be helpful to clarify functional role of these SNPs [9–11]. A 18 bp insertion/deletion (-2549) polymorphism was in absolute linkage disequilibrium (LD) with rs833061[25]. Other previous study also confirmed mentioning of complete linkage between the -2578 and -2549 polymorphisms, there was completely linked between -2447 deletion G and -2578 A allele; and -2578 A>C was chosen as marker for all completely linked polymorphism in this fragment [26].

According to our knowledge and Internet journal searching, there was no study that mentioned illness progression in chronic liver disease based on VEGF gene polymorphism studies. This study has concluded the occurrence of allele A and SNP A>C in DNA VEGF gene -2578 can predict illness progression in LC to HCC (allele A; *OR* 2.26) and healthy to CH/LC/HCC (SNP A>C, *OR* 1.14; allele A, *OR* 1.65; and SNP A>C, *OR* 1.41).

### Limitations

Our study is limited by a number of factors that require attention. First, the study design was neither cohort nor nested case-control study. As we know, disease progression of CH to LC or HCC needs a long time period (10–30 years), and most of the patients that come to the hospital are already in advanced liver condition. Multivariate and regression models using more variables (clinical and SNPs DNA genes) were needed to design a better model for prediction of illness progression. Secondly, a hospital-based study can’t reflect the real condition in the general population. Core biopsy was not done because approvals for invasive procedures and liver surgeries (liver resection or transplantation) were only rarely given, so we cannot examine the actual VEGF and VEGFR hepatocyte.

In conclusion, occurrence of allele A VEGF gene (-2587) and SNP A>C might predict illness progression from healthy to CH, LC or HCC and LC to HCC.

## Supporting information

S1 Table Supplement raw data. (DOCX)

## Abbreviations

5’UTR: 5’untranlated region
bp: base-pair
CH: chronic hepatitis
CLD: chronic liver disease
DNA: deoxyribo nucleic acid
EDTA: ethylene diamine tetra acetic acid
HCC: hepatocellular carcinoma
HIFs: hypoxia-inducible factors
HR: hazard ratio
Ht: haplotype
HWE: Hardy-Weinberg equilibrium
INR: International normalized ratio
LC: liver cirrhosis
liver-US: liver ultrasound
MSCT: multi-slice computed tomography
OR: odd ratio
*P*: significant value
rs: reference sequence
SNP: single nucleotide polymorphism
VEGF: vascular endothelial growth factor
VEGFR-2: VEGF receptor-2
