# Supplementary material for: Difference of polymorphism VEGF-gene rs699947 in Indonesian chronic liver disease population
Source: PLoS One. 2017 Aug 24;12(8):e0183503. doi: 10.1371/journal.pone.0183503 (PMC5570295; doi:10.1371/journal.pone.0183503)
Supplement: S1 Table — (DOCX) [file pone.0183503.s001.docx]

**S1 Table. Supplement Raw Data**

| **No** | **Age**  **(Year)** | **Gender** | **Diagnosis** | **Number of rs699947 (base pair)** | **GC content**  **(%)** | **AT content (%)** | **Genotype SNP -2578** | **Del -2549** | **I/D** | **Ht**  **CCGACCCC** |
| --- | --- | --- | --- | --- | --- | --- | --- | --- | --- | --- |
| 1 | 40 | M | CH | 258 | 55.42 | 44.57 | AC | No | Yes | Yes |
| 2 | 64 | M | LC | 259 | 55.59 | 44.40 | AC | No | Yes | No |
| 3 | 45 | M | LC | 240 | 55.41 | 44.58 | AA | Yes | No | No |
| 4 | 60 | M | LC | 244 | 54.50 | 45.49 | CC | Yes | No | No |
| 5 | 50 | M | HCC | 242 | 54.61 | 45.28 | AA | No | No | No |
| 6 | 64 | M | HCC | 262 | 55.72 | 44.27 | AA | No | No | No |
| 7 | 61 | F | LC | 259 | 55.21 | 44.78 | AC | No | Yes | Yes |
| 8 | 49 | M | LC | 242 | 54.95 | 45.04 | CC | Yes | No | No |
| 9 | 43 | F | LC | 258 | 55.03 | 44.96 | CA | No | Yes | No |
| 10 | 59 | M | HCC | 243 | 54.32 | 45.67 | CC | Yes | No | No |
| 11 | 47 | F | LC | 241 | 54.35 | 45.64 | CC | Yes | No | No |
| 12 | 68 | F | LC | 240 | 54.16 | 45.83 | CC | Yes | No | No |
| 13 | 46 | M | HCC | 241 | 54.35 | 45.64 | CC | Yes | No | No |
| 14 | 68 | F | LC | 242 | 55.37 | 44.62 | CC | Yes | No | No |
| 15 | 48 | M | CH | 240 | 55.00 | 45.00 | CC | Yes | No | No |
| 16 | 42 | F | CH | 240 | 55.41 | 44.58 | CC | Yes | No | No |
| 17 | 28 | M | CH | 258 | 55.03 | 44.96 | AA | No | No | No |
| 18 | 40 | M | CH | 258 | 53.65 | 45.34 | AC | No | Yes | No |
| 19 | 60 | F | HCC | 258 | 54.26 | 45.73 | AC | No | Yes | No |
| 20 | 47 | M | HCC | 241 | 54.77 | 45.22 | CA | Yes | No | No |
| 21 | 22 | M | LC | 244 | 55.32 | 44.67 | CC | Yes | No | No |
| 22 | 52 | M | HCC | 259 | 54.44 | 45.55 | AC | No | Yes | No |
| 23 | 42 | M | LC | 244 | 54.91 | 45.08 | CC | Yes | No | No |
| 24 | 60 | F | CH | 246 | 54.87 | 45.12 | CA | No | Yes | No |
| 25 | 52 | M | LC | 243 | 55.15 | 44.85 | CC | Yes | No | No |
| 26 | 50 | M | LC | 259 | 55.59 | 44.40 | CA | No | Yes | No |
| 27 | 63 | F | LC | 258 | 55.81 | 44.18 | CA | No | Yes | No |
| 28 | 41 | M | LC | 258 | 55.42 | 44.57 | AC | No | Yes | No |
| 29 | 30 | F | CH | 243 | 54.32 | 45.67 | CC | Yes | No | No |
| 30 | 48 | M | HCC | 260 | 55.76 | 44.23 | AC | No | Yes | No |
| 31 | 51 | M | HCC | 242 | 54.95 | 45.04 | CC | Yes | No | No |
| 32 | 27 | M | LC | 241 | 54.35 | 45.64 | CC | Yes | No | No |
| 33 | 57 | M | LC | 259 | 55.21 | 44.78 | CA | No | Yes | No |
| 34 | 55 | F | LC | 241 | 55.60 | 44.39 | CC | Yes | No | No |
| 35 | 72 | F | LC | 241 | 54.77 | 45.22 | CC | Yes | No | No |
| 36 | 33 | F | HCC | 241 | 55.18 | 44.81 | CC | Yes | No | No |
| 37 | 43 | F | LC | 262 | 55.34 | 44.65 | CA | No | Yes | No |
| 38 | 62 | M | LC | 258 | 54.26 | 45.73 | AA | No | No | No |
| 39 | 63 | M | HCC | 242 | 55.37 | 44.62 | CC | Yes | No | No |
| 40 | 47 | M | LC | 243 | 54.32 | 45.67 | CC | Yes | No | No |
| 41 | 52 | F | CH | 257 | 54.86 | 45.13 | AC | No | Yes | Yes |
| 42 | 51 | M | LC | 241 | 54.77 | 45.22 | CC | Yes | No | No |
| 43 | 53 | F | HCC | 255 | 56.47 | 43.52 | CA | No | Yes | No |
| 44 | 48 | F | LC | 244 | 55.32 | 44.67 | CC | Yes | No | No |
| 45 | 48 | F | CH | 258 | 54.26 | 45.73 | CA | No | Yes | No |
| 46 | 58 | F | LC | 242 | 54.54 | 45.45 | CC | Yes | No | No |
| 47 | 58 | M | HCC | 244 | 55.32 | 44.67 | CC | Yes | No | No |
| 48 | 57 | M | LC | 257 | 54.86 | 45.13 | AC | No | Yes | No |
| 49 | 55 | M | LC | 245 | 55.10 | 44.89 | CC | Yes | No | No |
| 50 | 45 | M | LC | 241 | 54.35 | 45.64 | CC | Yes | No | No |
| 51 | 61 | M | CH | 244 | 54.91 | 45.08 | CC | Yes | No | No |
| 52 | 60 | M | LC | 260 | 55.38 | 44.61 | CA | No | Yes | No |
| 53 | 67 | M | HCC | 262 | 56.48 | 43.51 | AA | No | No | No |
| 54 | 65 | M | LC | 259 | 55.59 | 44.40 | CA | No | Yes | No |
| 55 | 78 | M | HCC | 243 | 55.14 | 44.85 | CC | Yes | No | No |
| 56 | 62 | M | LC | 242 | 54.54 | 45.45 | CC | Yes | No | No |
| 57 | 54 | M | LC | 256 | 55.07 | 44.92 | CA | No | Yes | No |
| 58 | 67 | M | LC | 242 | 54.54 | 45.45 | CC | Yes | No | No |
| 59 | 59 | F | CH | 244 | 54.50 | 45.49 | CC | Yes | No | No |
| 60 | 54 | M | CH | 257 | 54.47 | 45.52 | AC | No | Yes | No |
| 61 | 55 | F | CH | 240 | 54.58 | 45.41 | CC | Yes | No | No |
| 62 | 60 | M | CH | 244 | 53.27 | 46.72 | CC | Yes | No | No |
| 63 | 68 | F | CH | 259 | 54.82 | 45.17 | AC | No | Yes | No |
| 64 | 38 | F | CH | 258 | 56.20 | 43.79 | CA | No | Yes | No |
| 65 | 40 | F | CH | 260 | 55.00 | 45.00 | AC | No | Yes | No |
| 66 | 40 | M | CH | 259 | 54.44 | 45.55 | AC | No | No | No |
| 67 | 45 | F | CRTL | 242 | 55.37 | 44.62 | CC | Yes | No | No |
| 68 | 48 | M | CTRL | 244 | 54.91 | 45.08 | CA | Yes | No | No |
| 69 | 55 | M | CTRL | 240 | 54.58 | 45.41 | CC | Yes | No | No |
| 70 | 43 | F | CTRL | 242 | 56.19 | 43.80 | CC | Yes | No | No |
| 71 | 69 | M | CTRL | 260 | 55.00 | 45.00 | AC | No | No | No |
| 72 | 44 | F | CTRL | 244 | 54.91 | 45.08 | CC | Yes | No | No |
| 73 | 53 | M | CTRL | 241 | 54.77 | 45.22 | CC | Yes | No | No |
| 74 | 36 | M | CTRL | 261 | 54.78 | 45.21 | AA | No | No | No |
| 75 | 63 | F | CTRL | 261 | 55.17 | 44.82 | AC | No | Yes | No |
| 76 | 40 | M | CTRL | 244 | 56.55 | 43.44 | CC | Yes | No | No |
| 77 | 48 | F | CTRL | 258 | 55.42 | 44.57 | AC | No | Yes | No |
| 78 | 43 | M | CTRL | 259 | 55.21 | 44.78 | AC | No | Yes | Yes |
| 79 | 29 | M | CTRL | 259 | 55.21 | 44.78 | AC | No | Yes | Yes |
| 80 | 52 | M | CTRL | 258 | 54.65 | 45.34 | AA | No | No | No |
| 81 | 53 | F | CTRL | 260 | 55.38 | 44.61 | AC | No | Yes | No |
| 82 | 55 | F | CTRL | 256 | 55.85 | 44.14 | CA | No | Yes | No |
| 83 | 41 | M | CTRL | 243 | 55.14 | 44.85 | CC | Yes | No | No |
| 84 | 50 | M | CTRL | 259 | 55.59 | 44.40 | CA | No | Yes | No |
| 85 | 35 | M | CTRL | 259 | 54.82 | 45.17 | AC | No | Yes | No |
| 86 | 53 | M | CTRL | 258 | 55.03 | 44.96 | AC | No | Yes | Yes |
| 87 | 36 | M | CTRL | 259 | 55.98 | 44.01 | CA | No | Yes | No |
| 88 | 66 | M | CTRL | 221 | 55.14 | 44.85 | CC | Yes | No | No |
| 89 | 36 | M | HCC | 259 | 55.59 | 44.40 | AC | No | Yes | No |
| 90 | 52 | M | CTRL | 258 | 55.03 | 44.96 | AC | No | Yes | Yes |
| 91 | 35 | M | CTRL | 257 | 54.86 | 45.13 | AC | No | Yes | Yes |
| 92 | 66 | F | CTRL | 238 | 55.46 | 44.53 | CC | Yes | No | No |
| 93 | 67 | M | CTRL | 243 | 54.73 | 45.26 | CC | Yes | No | No |
| 94 | 52 | M | HCC | 243 | 54.73 | 45.26 | CC | Yes | No | No |
| 95 | 60 | F | CTRL | 243 | 54.73 | 45.26 | CC | Yes | No | No |
| 96 | 53 | M | CH | 244 | 54.50 | 45.49 | CC | Yes | No | No |
| 97 | 57 | M | HCC | 261 | 55.17 | 44.82 | AC | No | Yes | Yes |
| 98 | 38 | F | HCC | 259 | 55.59 | 44.4 | CA | No | Yes | Yes |
| 99 | 46 | F | CH | 259 | 54.82 | 45.17 | AC | No | Yes | Yes |
| 100 | 57 | M | HCC | 257 | 55.25 | 44.74 | CA | No | Yes | No |
| 101 | 66 | M | LC | 244 | 54.50 | 45.49 | CC | Yes | No | No |
| 102 | 62 | M | CH | 243 | 55.14 | 44.85 | CC | Yes | No | No |
| 103 | 73 | F | CH | 242 | 56.19 | 43.80 | CC | Yes | No | No |
| 104 | 65 | M | HCC | 244 | 55.32 | 44.67 | AC | No | Yes | Yes |
| 105 | 42 | M | CH | 260 | 56.53 | 43.46 | CC | Yes | No | No |
| 106 | 38 | M | HCC | 259 | 55.59 | 44.40 | CA | No | Yes | No |
| 107 | 57 | F | HCC | 243 | 55.14 | 44.85 | CC | Yes | No | No |
| 108 | 35 | M | HCC | 261 | 53.78 | 45.21 | AA | Yes | No | No |
| 109 | 46 | M | HCC | 259 | 54.82 | 45.17 | AC | Yes | No | No |
| 110 | 58 | F | HCC | 242 | 54.95 | 45.04 | CC | Yes | No | No |
| 111 | 59 | F | CH | 248 | 53.62 | 46.37 | CA | No | Yes | No |
| 112 | 28 | F | CH | 247 | 53.84 | 46.15 | CA | Yes | No | No |
| 113 | 59 | M | HCC | 258 | 55.81 | 44.18 | AA | No | No | No |
| 114 | 28 | F | HCC | 243 | 54.32 | 45.67 | CC | Yes | No | No |
| 115 | 58 | F | HCC | 217 | 53.94 | 46.05 | CC | Yes | No | No |
| 116 | 66 | M | CH | 243 | 54.73 | 45.26 | AC | No | Yes | Yes |
| 117 | 63 | M | LC | 241 | 55.60 | 44.39 | CC | Yes | No | No |
| 118 | 45 | F | HCC | 260 | 54.61 | 45.38 | AC | No | Yes | Yes |
| 119 | 28 | F | HCC | 244 | 54.50 | 45.49 | AC | No | Yes | Yes |
| 120 | 76 | M | CH | 262 | 56.10 | 43.89 | CA | No | Yes | Yes |
| 121 | 55 | F | LC | 260 | 55.00 | 45.00 | AC | No | No | No |
| 122 | 68 | M | HCC | 240 | 53.75 | 46.25 | CC | Yes | No | No |
| 123 | 22 | F | CH | 244 | 55.32 | 44.67 | CA | No | Yes | No |
| 124 | 55 | F | HCC | 256 | 54.29 | 45.70 | CA | No | Yes | No |
| 125 | 20 | M | CH | 257 | 54.86 | 45.13 | AC | No | No | No |
| 126 | 67 | M | HCC | 261 | 55.17 | 44.2 | CA | No | Yes | No |
| 127 | 49 | M | CH | 244 | 54.09 | 45.90 | AC | No | Yes | No |
| 128 | 77 | F | HCC | 243 | 55.14 | 44.85 | CA | Yes | No | No |
| 129 | 56 | M | LC | 258 | 55.03 | 44.96 | CA | No | Yes | No |
| 130 | 39 | M | HCC | 242 | 54.94 | 45.04 | CC | Yes | No | No |
| 131 | 32 | M | CH | 259 | 55.59 | 44.04 | CA | No | Yes | No |
| 132 | 60 | F | CH | 259 | 55.21 | 44.78 | AC | No | Yes | No |
| 133 | 45 | F | CH | 243 | 55.14 | 44.85 | CC | Yes | No | No |
| 134 | 48 | M | HCC | 243 | 55.96 | 44.03 | CC | Yes | No | No |
| 135 | 61 | F | CH | 245 | 54.28 | 45.71 | CA | Yes | No | No |
| 136 | 57 | M | HCC | 259 | 55.59 | 44.40 | CA | No | Yes | No |
| 137 | 59 | F | CH | 261 | 54.78 | 45.21 | AA | No | No | No |
| 138 | 32 | M | HCC | 241 | 55.18 | 44.81 | CC | Yes | No | No |
| 139 | 40 | M | HCC | 246 | 54.87 | 45.12 | CA | Yes | No | No |
| 140 | 60 | M | HCC | 260 | 55.76 | 44.23 | CA | No | Yes | No |
| 141 | 80 | M | HCC | 257 | 54.47 | 45.52 | CA | No | Yes | No |
| 142 | 40 | M | CH | 241 | 53.52 | 46.47 | CC | Yes | No | No |
| 143 | 41 | M | HCC | 240 | 54.16 | 45.83 | CC | Yes | No | No |
| 144 | 30 | M | HCC | 242 | 54.95 | 45.04 | CC | Yes | No | No |
| 145 | 55 | M | CH | 261 | 54.78 | 45.21 | AC | No | Yes | No |
| 146 | 64 | M | LC | 258 | 54.26 | 45.73 | CA | No | Yes | No |
| 147 | 45 | F | CH | 260 | 55.00 | 45.00 | CA | No | Yes | No |
| 148 | 26 | F | CH | 240 | 55.41 | 44.58 | CC | Yes | No | No |
| 149 | 70 | F | LC | 243 | 53.90 | 46.09 | CC | Yes | No | No |
| 150 | 39 | M | HCC | 264 | 54.92 | 45.07 | AC | No | Yes | No |
| 151 | 51 | F | CTRL | 258 | 55.42 | 44.57 | AC | No | Yes | No |
| 152 | 45 | F | CTRL | 260 | 54.61 | 45.38 | AA | No | No | No |
| 153 | 45 | F | CTRL | 243 | 53.90 | 46.09 | CC | Yes | No | No |
| 154 | 45 | F | CTRL | 243 | 54.73 | 45.26 | CC | Yes | No | No |
| 155 | 29 | F | CTRL | 260 | 55.00 | 45.00 | AC | No | Yes | Yes |
| 156 | 45 | F | CTRL | 259 | 54.82 | 45.17 | AC | No | Yes | No |
| 157 | 46 | F | CTRL | 243 | 54.73 | 45.26 | CC | Yes | No | No |
| 158 | 29 | F | CTRL | 257 | 54.86 | 45.13 | AC | No | Yes | No |
| 159 | 24 | F | CTRL | 241 | 53.94 | 46.05 | CC | Yes | No | No |
| 160 | 24 | F | CTRL | 256 | 55.46 | 44.53 | AC | No | Yes | Yes |
| 161 | 51 | F | CTRL | 242 | 54.54 | 45.45 | CC | Yes | No | No |
| 162 | 27 | M | CTRL | 261 | 55.55 | 44.44 | AA | No | No | No |
| 163 | 46 | M | CTRL | 258 | 54.65 | 45.34 | AC | No | Yes | No |
| 164 | 25 | M | CTRL | 258 | 55.03 | 44.96 | AC | No | Yes | Yes |
| 165 | 32 | M | CTRL | 242 | 53.71 | 46.26 | CC | Yes | No | No |
| 166 | 31 | F | CTRL | 256 | 55.85 | 44.14 | CA | No | Yes | No |
| 167 | 28 | M | CTRL | 243 | 54.32 | 45.67 | CC | Yes | No | No |
| 168 | 24 | M | CTRL | 259 | 54.82 | 45.17 | AC | No | Yes | No |
| 169 | 24 | F | CTRL | 257 | 54.08 | 45.91 | AC | No | Yes | No |
| 170 | 28 | M | CTRL | 242 | 53.30 | 46.69 | CC | Yes | No | No |
| 171 | 24 | M | CTRL | 240 | 55.00 | 45.00 | CC | Yes | No | No |
| 172 | 26 | F | CTRL | 264 | 55.03 | 44.69 | AA | No | No | No |
| 173 | 30 | F | CTRL | 242 | 55.78 | 44.21 | CC | Yes | No | No |
| 174 | 30 | M | CTRL | 260 | 55.00 | 45.00 | AC | No | Yes | No |
| 175 | 24 | M | CTRL | 258 | 54.65 | 45.34 | AC | No | Yes | No |
| 176 | 29 | M | CTRL | 258 | 55.42 | 44.47 | CA | No | Yes | No |
| 177 | 24 | M | CTRL | 244 | 53.27 | 46.72 | CC | Yes | No | No |
| 178 | 29 | F | CTRL | 261 | 55.17 | 44.82 | AC | No | Yes | No |
| 179 | 23 | F | CTRL | 260 | 54.23 | 45.76 | AA | No | No | No |
| 180 | 25 | F | CTRL | 242 | 54.54 | 45.45 | CC | Yes | No | No |
| 181 | 23 | M | CTRL | 254 | 54.72 | 45.27 | AC | No | Yes | No |
| 182 | 21 | M | CTRL | 260 | 55.38 | 44.61 | AC | No | Yes | No |
| M, male; F, female; HCC, hepatocelular carcinoma; LC, liver cirrhotic; CH, chronic hepatitis; CRTL, control healthy subject  Del,deletion 18bp at -2549; I/D, insertion and/or deletion; Ht, haplotype | | | | | | | | | | |
